# Structural basis of *Pseudomonas aeruginosa* penicillin binding protein 3 inhibition by the siderophore-antibiotic cefiderocol†

**DOI:** 10.1039/d4sc04937c

**Published:** 2024-09-18

**Authors:** Helen G. Smith, Shyam Basak, Victor Aniebok, Matthew J. Beech, Faisal M. Alshref, Mark D. Allen, Alistair J. M. Farley, Christopher J. Schofield

**Affiliations:** a Department of Chemistry, University of Oxford 12 Mansfield Road Oxford OX1 3TA UK christopher.schofield@chem.ox.ac.uk; b Ineos Oxford Institute for Antimicrobial Research, University of Oxford 12 Mansfield Road Oxford OX1 3TA UK; c Department of Biochemistry, Faculty of Science, King AbdulAziz University Jeddah Saudi Arabia

## Abstract

The breakthrough cephalosporin cefiderocol, approved for clinical use in 2019, has activity against many Gram-negative bacteria. The catechol group of cefiderocol enables it to efficiently enter bacterial cells *via* the iron/siderophore transport system thereby reducing resistance due to porin channel mutations and efflux pump upregulation. Limited information is reported regarding the binding of cefiderocol to its key proposed target, the transpeptidase penicillin binding protein 3 (PBP3). We report studies on the reaction of cefiderocol and the related cephalosporins ceftazidime and cefepime with *Pseudomonas aeruginosa* PBP3, including inhibition measurements, protein observed mass spectrometry, and X-ray crystallography. The three cephalosporins form analogous 3-exomethylene products with *P. aeruginosa* PBP3 following elimination of the C3′ side chain. pIC_50_ and *k*_inact_/*K*_i_ measurements with isolated PBP3 imply ceftazidime and cefiderocol react less efficiently than cefepime and, in particular, meropenem with *P. aeruginosa* PBP3. Crystal structures inform on conserved and different interactions involved in binding of the three cephalosporins and meropenem to *P. aeruginosa* PBP3. The results will aid development of cephalosporins with improved PBP3 inhibition properties.

## Abstract 2

The cephalosporin antibiotics cefiderocol, ceftazidime and cefepime undergo fragmentation on reaction with penicillin binding protein 3 from *Pseudomonas aeruginosa.*

## Graphical abstract

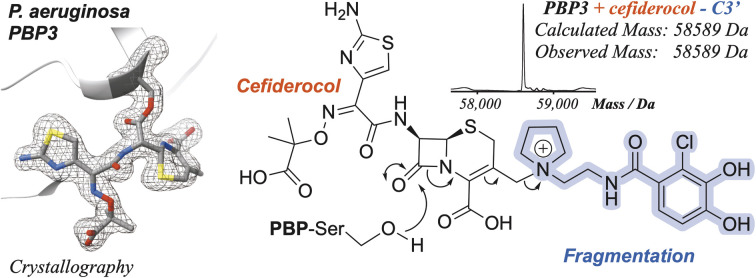

## Introduction

The cephalosporin cefiderocol (Fetroja©), which was developed by Shionogi, is a pioneering example of a ‘Trojan horse’ class antibiotic: its C3′ linked catechol group acts as a siderophore enabling it to efficiently enter Gram-negative cells *via* the iron/siderophore transport system.^1,2^ Cefiderocol is thus less susceptible to resistance mechanisms which affect other related antibiotics, in particular mutations in porin channels and upregulation of efflux pumps.^1–3^ The C3 and C7 sidechains of cefiderocol (Table 1) help to provide stability to serine β-lactamase (SBL) mediated resistance *via* β-lactam hydrolysis.^2,4^ Cefiderocol is active against multiple multi-drug resistant Gram-negative pathogens, including *Klebsiella pneumoniae*, *Pseudomonas aeruginosa*, *Escherichia coli* and *Acinetobacter baumannii*, which are on the World Health Organisation's list of priority pathogens, for which development of novel therapeutics is urgently required.^1,2,4,5^

**Table 1:** Inhibition of *P. aeruginosa* PBP3 by cephalosporins

Compound	pIC_50_ (S2d, *n* = 3)^a^	pIC_50_ (bocillin, *n* = 3)^b^	*k*_inact_/*K*_i_ (M^−1^ s^−1^, *n* = 3)^c^	Inhibitor structure	Complex formed on PBP binding	Eliminated on binding
Cefiderocol	7.3 ± 0.1	7.0 ± 0.1	3000 ± 200	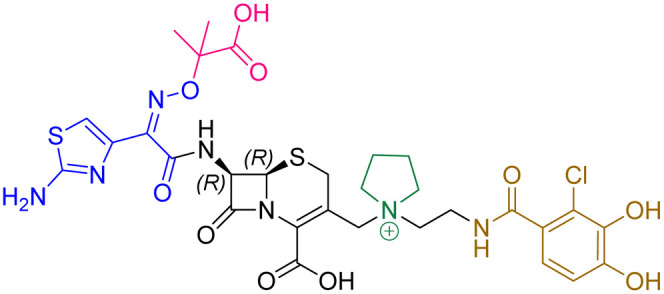	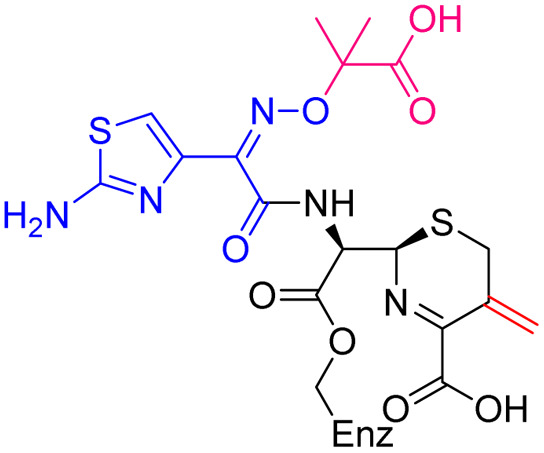	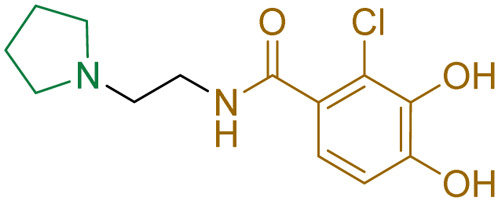
Ceftazidime	7.1 ± 0.1	6.6 ± 0.1	3400 ± 400	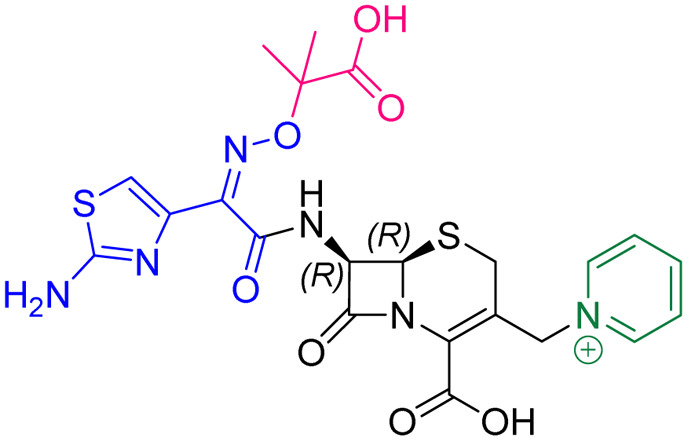	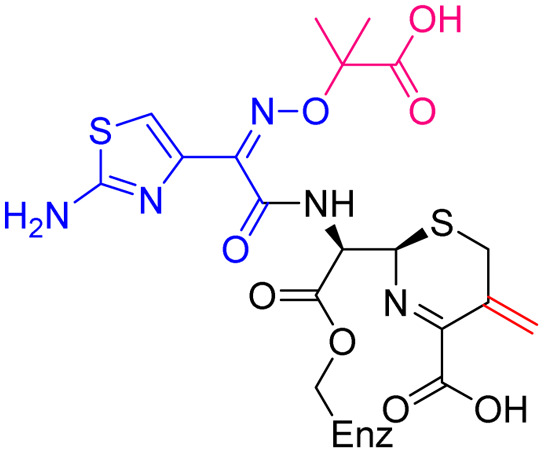	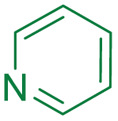
Cefepime	7.4 ± 0.2	7.0 ± 0.1	9600 ± 2100	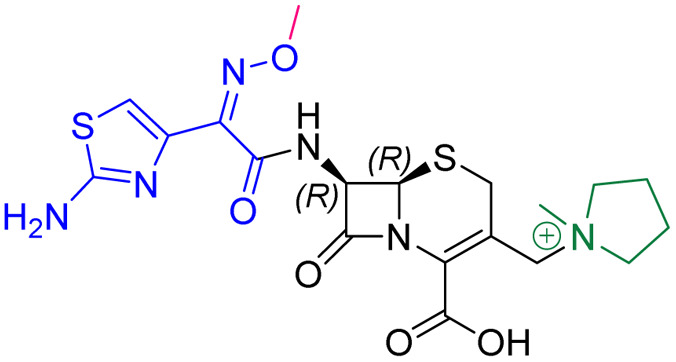	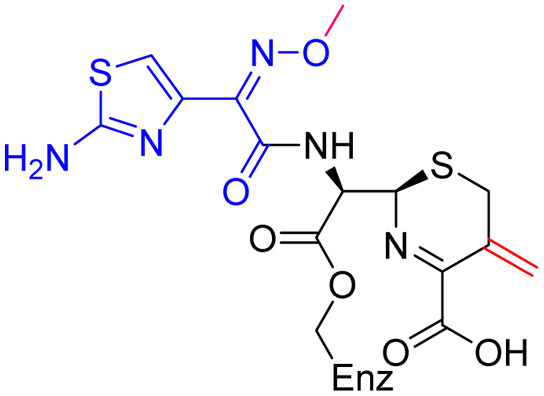	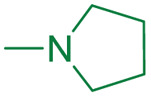
Meropenem	7.2 ± 0.2	7.4 ± 0.1	11 000 ± 3900	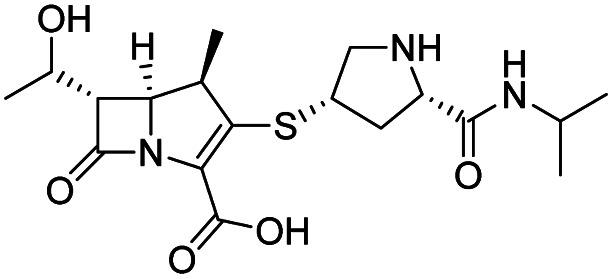	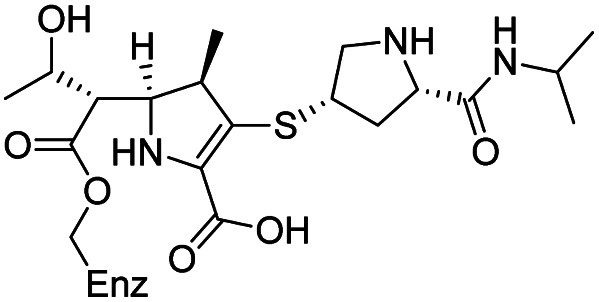	N/A

a Using 300 nM PBP3, 1.5 mM S2d and 0.5 mM mBBr.

b Using 311 nM PBP3 and 30 nM Bocillin-FL.

c Using 60 nM PBP3 and 30 nM Bocillin-FL. All measurements are reported as the mean average ± standard error of three independent experiments (each composed of technical triplicates). Meropenem was included as a control. Compounds are coloured according to their conserved functional groups including; C7 oxime (blue), C3 pyrrolidinium or pyridinium (green), chlorocatechol (brown), oxime carboxylic acid/methyl (pink), exomethylene (red). Note cefiderocol and ceftazidime have identical C7 side chains.

Despite cefiderocol being approved by the United States Food and Drug Administration (FDA) (and other agencies) for treatment of complicated urinary tract infections, hospital-acquired bacterial pneumoniae and ventilator-associated bacterial pneumoniae in 2019, limited information about its interaction with its key proposed bacterial cell wall target, the transpeptidase penicillin binding protein 3 (PBP3), has been reported.^6^ PBP3 catalyses the formation of crosslinks between peptidoglycan strands within the bacterial cell wall^7,8^ and is part of the divisome complex, playing an essential role in cell division.^9–11^ Inhibition of PBP3 elicits transcription of genes involved in the SOS response, ultimately leading to cell cycle arrest.^8^ Consequently, PBP3 is one of the most validated and clinically important antibacterial targets.^12^

The lack of information regarding the binding of cefiderocol to PBP3 motivated us to undertake structural and inhibition studies, comparing the results with those for the structurally related cephalosporin antibiotics cefepime and ceftazidime. Co-crystallisation studies of the three cephalosporins with *P. aeruginosa* PBP3 (hereafter PBP3) reveals their binding mode and active site interactions. Inhibition measurements and protein-observed mass spectrometry reveals differences in the way these compounds react with PBP3, information that will be useful in the design of improved PBP3 inhibitors.

## Results and discussion

### Cefiderocol, ceftazidime and cefepime are potent inhibitors of *P. aeruginosa* PBP3

PBP3 transpeptidases are proposed to be key targets for the cephalosporins and most other β-lactam antibiotics.^13^ We thus investigated the effects of cefiderocol, cefepime and ceftazidime as well as the carbapenem meropenem on purified recombinant PBP3 from *P. aeruginosa*, which was prepared *via* a modification of the reported procedure.^14^ pIC_50_ values against PBP3 were measured using two complementary assay methods (Table 1). In one method, the thioester substrate analogue S2d is hydrolysed by PBP3 (ESI Scheme S1†) releasing d-alanine and a free thiol.^15,16^ The latter reacts with monobromobimane (mBBr), a fluorescent dye, enabling measurement of the turnover rate and determination of pIC_50_ values. The second method employed used Bocillin-FL, a fluorescent penicillin analogue, which covalently modifies the active site of the PBP, producing a fluorescence polarisation readout (ESI Scheme S2†).^17,18^ Whilst the pIC_50_ measurements obtained using the S2d assay were greater than those obtained using Bocillin-FL, the values measured using both methods followed the same trend.

Using both methods, cefiderocol and cefepime were found to be more potent inhibitors of *P. aeruginosa* PBP3 than ceftazidime and meropenem, though the differences between the assay results were small. Consequently, a third assay was undertaken utilising Bocillin-FL to measure *k*_inact_/*K*_i_ values by monitoring binding to the target PBP in the presence of varying concentrations of each cephalosporin inhibitor.^19,20^*k*_inact_/*K*_i_ values reflect both the reactivity (*k*_inact_) and binding affinity (*K*_I_), therefore better encapsulate the time-dependent kinetics of covalently reacting inhibitors, information which is not obtained by measurement of pIC_50_ values.^19,20^ The *k*_inact_/*K*_i_ of cefepime was slightly less than that of meropenem (9600 and 11 000 M^−1^ s^−1^, respectively), whilst the *k*_inact_/*K*_i_ values of cefiderocol and ceftazidime were similar and substantially lower (3000 and 3400 M^−1^ s^−1^, respectively). The results thus indicate that with these turnover assays under our assay conditions, albeit using unnatural substrates, cefepime and meropenem are more potent inhibitors of isolated *P. aeruginosa* PBP3 than either cefiderocol or ceftazidime.

### A competitive binding experiment demonstrates an order of activity of the cephalosporins

**Fig. 1:**
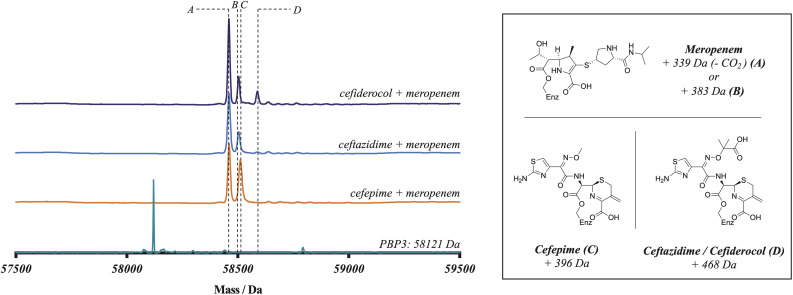
Reaction of *P. aeruginosa* PBP3 with cephalosporins in competition with meropenem (1 : 1 ratio) informs on the efficiency of reaction. Electrospray-ionisation quadrupole time-of-flight (ESI-Q-TOF) mass spectra imply that cefepime (C) reacts more efficiently with PBP3 than cefiderocol (D), which reacts more efficiently than ceftazidime (D). The spectra shown are representative of three technical repeats.

To further probe the relative rates of reaction of the four β-lactams with PBP3, a competitive binding experiment employing PBP3-observed mass spectrometry (MS) was developed. The three cephalosporins were prepared in one-to-one-mixtures with meropenem, prior to addition to a PBP3 solution. Note, the mass of meropenem is sufficiently different from those of cephalosporins to enable the PBP3 complex masses to be differentiated. Following 10 minutes co-incubation with PBP3, the reactions were quenched with formic acid and analysed by protein-observed MS (under denaturing conditions) (Fig. 1). As meropenem is known to bind efficiently and likely irreversibly to PBP3 ^14^ this method enables the rates of binding of the cephalosporins to the target protein to be investigated. Notably, following mixing we saw no evidence for a reduction in the intensity of the PBP3-inhibitor complex peaks over the time course of our analyses (10 min), implying (near) irreversible inhibition. Coincubation of cefepime with meropenem produced an ∼1 : 1 mixture of cefepime and meropenem modified PBP, indicating that cefepime binds almost as rapidly to *P. aeruginosa* PBP3 as does meropenem, consistent with the *k*_inact_/*K*_I_ measurements. With cefiderocol, a small amount of cefiderocol modified PBP3 was observed, however, most of the observed complex was meropenem modified PBP3. With ceftazidime, the product formed was exclusively meropenem-modified PBP3. The combined results of the meropenem competition analyses indicate that of the three investigated cephalosporins, cefepime reacts covalently most efficiently with isolated PBP3 and with a similar efficiency to meropenem, followed by cefiderocol, with ceftazidime being the least efficient. Since cefiderocol and ceftazidime have the same C7 side chain, the results imply both C7 and C3 cephalosporin side chains are involved in determining binding efficiency.

### X-ray crystal structures of *P. aeruginosa* PBP3 in complex with cefiderocol, cefepime, ceftazidime, and meropenem identify key active site interactions

**Fig. 2:**
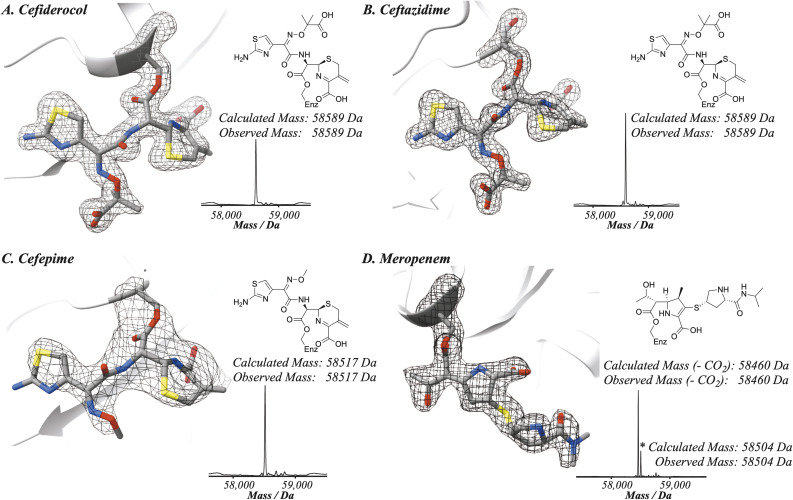
Reaction of cephalosporins with the nucleophilic serine of *P. aeruginosa* PBP3 proceeds with elimination of the C3 side chain. Evidence for elimination is provided by X-ray crystallography and protein-observed mass spectrometry of *P. aeruginosa* PBP3 following reaction with; (A) cefiderocol, (mF_o_-DF_c_ polder OMIT map contoured at 5.82*σ*), (B) ceftazidime, (mF_o_-DF_c_ polder OMIT map contoured at 5.00*σ*), (C) cefepime, (mF_o_-DF_c_ polder OMIT map contoured at 3.30*σ*) and (D) meropenem, (mF_o_-DF_c_ polder OMIT map contoured at 4.68*σ*). In each case the deconvoluted mass spectra following reaction of the cephalosporins with *P. aeruginosa* PBP3 support elimination of the C3 side chain from the cephalosporin.

**Fig. 3:**
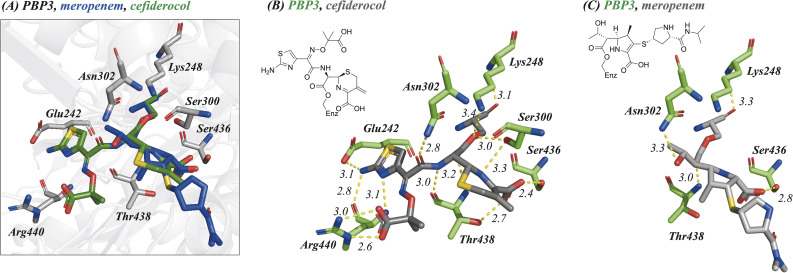
Crystallographic studies indicate that cephalosporins react with the active site serine of *P. aeruginosa* PBP3 resulting in elimination of the C3 group. (A) Superimposition of *P. aeruginosa* PBP3 active site following reaction with cefiderocol (green, PDB:9FZ7) and meropenem (blue, PDB:9FZE). Interaction map displaying polar interactions formed between (B) cefiderocol (PDB:9FZ7), (C) meropenem (PDB:9FZE) and residues within the *P. aeruginosa* PBP3 active site. Compounds are coloured grey, interacting residues are green. Distances are in Å.

To compare the binding modes of cefiderocol, cefepime, ceftazidime and meropenem with PBP3, a truncated construct more amenable to crystallography was generated. Structures of the new PBP3 construct with the antibiotics bound were obtained *via* co-crystallisation (Fig. 2 and 3, ESI Fig. S1–S4†). We also obtained a structure of apo-PBP3 for comparison. The structures were obtained in the *P*2_1_2_1_2_1_ space group with one molecule per asymmetric unit and diffracted to resolutions of 1.80 Å (cefiderocol), 2.70 Å (cefepime), 1.80 Å (ceftazidime), 2.10 Å (meropenem), and 2.11 Å (apo-PBP3) (ESI Table S1†). Previously, a structure of PBP3 complexed with ceftazidime has been reported, this was also obtained in the *P*2_1_2_1_2_1_ space group with one molecule per asymmetric unit.^7^

The PBP3 fold is composed of two domains: a C-terminal transpeptidase domain which catalyses crosslinking of the pentapeptide strands of the lipid II subunits of peptidoglycan, and an N-terminal ‘dimerisation’-domain, comprised of pedestal and anchor sub-domains, that enables interactions with other proteins within the divisome complex (ESI, Fig. S4†).^7,9,11^ There is little variation in the overall fold of PBP3 in the structures with RMSD values compared to the apo-PBP3 of 1.04 Å (cefiderocol), 1.02 Å (cefepime), 1.07 Å (ceftazidime) and 0.29 Å (meropenem) (ESI, Table S2†). Note that our structure of PBP3 with ceftazidime is very similar to that reported (RMSD 1.54 Å).^7^ The transpeptidase domain, which is fully modified at its nucleophilic active site serine (Ser245) by reaction with the inhibitors, does not vary in fold or conformation across any of the structures (RMSD < 1.5 Å).

**Scheme 1:**
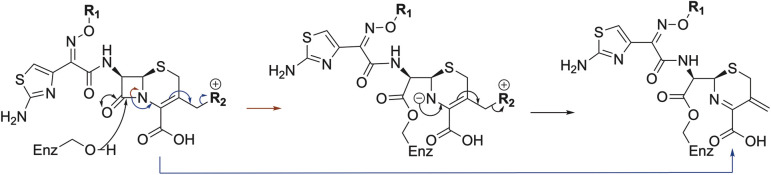
Outline mechanism for reaction of *P. aeruginosa* PBP3 with pyrrolidinium cephalosporins resulting in elimination of the C3 group. Reaction may either be concerted (blue arrows) or proceed *via* an anionic intermediate (red arrows). Elimination of the C3 side chain from cefiderocol produces an identical PBP3-bound complex to that generated on ceftazidime binding (Fig. 2).

As anticipated, all five antibiotics react with the nucleophilic serine (Ser245) to form acyl enzyme complexes, wherein the β-lactam derived carbonyl oxygen is positioned to hydrogen bond with the backbone NH of Thr438 (3.0–3.4 Å).^14,21^ Also consistent with previous studies and our protein-observed MS studies (Fig. 2 and ESI, Fig. S5†), in the crystal structures with the cephalosporins loss of the C3′ side chain is observed giving the exomethylene products (Scheme 1).^14^ By contrast the meropenem carbon scaffold remains intact (Fig. 2).^14^

Inspection of the residues in the active sites of the crystal structures (Fig. 3, ESI Fig. S1 and S2†) enables identification of residues involved in conserved interactions with the three cephalosporin derived complexes, in particular with Glu242, Ser300, Asn302, Ser436, Thr348, and Arg440.

One of the C4 carboxylic acid oxygens of the cephalosporins is positioned to form a single interaction with the hydroxyl group of Thr438 (O_cefiderocol_–O_Thr438_: ∼2.7 Å) and the other C4 carboxylic acid oxygen is positioned to interact with Ser436 (O_cefiderocol_–O_Ser436_: ∼2.4 Å) (Fig. 3, ESI Fig. S1 and S2†). In the case of cefepime, a further interaction between a C4 carboxylic acid oxygen and Ser300 also occurs (O_cefepime_–O_Ser300_: ∼3.1 Å). In the case of meropenem, its C3 carboxylic acid is only positioned to interact with Ser436 (O_meropenem_–O_Ser436_: ∼2.8 Å); Thr438 is more distant from the carboxylate (O_meropenem_–O_Thr438_: ∼4.1 Å) than with the cephalosporins (Fig. 3, ESI Fig. S1 and S2†).^14^

With the cephalosporin complex structures, the C7 amide oxygen is positioned to interact *via* a hydrogen bond with the side chain amide NH_2_ of Asn302 (∼2.8–3.0 Å). The C7 amide NH interacts with the backbone carbonyl of Thr438 (∼2.9–3.0 Å) in all the cephalosporin complex structures, as anticipated.^14^ With meropenem the alcohol of the C6 hydroxyethyl group makes an analogous interaction with Asn302 (see below).

The oxime-derivative side chain of the cephalosporins is positioned to interact with the carboxylic acid of Glu242, and, in particular with Arg440 (Fig. 3, ESI Fig. S1 and S2†). Notably, the O-methylated C7 oxime of cefepime and the identical carboxylic acid linked oximes of both cefiderocol/ceftazidime interact differently with Arg440. The C7 oxime linked carboxylic acids of cefiderocol/ceftazidime bind identically, directly interacting with the guanidino group of Arg440, being positioned to form two salt bridges (∼2.6–3.4 Å). Salt bridges with the guanidino group of Arg440 are absent in the cefepime bound structure; the C7 oxime is not observed to form any interactions with residues within the active site (Fig. 3, ESI Fig. S1 and S2†). The C7 aminothiazole moiety appears to form identical interactions in both the cefepime and cefiderocol/ceftazidime structures. The aminothiazole amine NH_2_ forms hydrogen bonds with both the side chain carbonyl oxygen of Glu242 (∼3.1–3.6 Å) and the backbone carbonyl oxygen of Arg440 (∼2.8–2.9 Å). The nitrogen atom of the aminothiazole ring also forms a hydrogen bond with the backbone amide NH_2_ of Arg440 (∼3.0–3.2 Å) (Fig. 3, ESI Fig. S1 and S2†).

In comparison with the meropenem bound structure, the cephalosporins form direct interactions with six residues within the active site, whilst meropenem interacts *via* direct polar interactions with only three residues (Asn302, Ser436, and Thr438).^14^ Further, meropenem is positioned to make only a single interaction with each of these three residues (Fig. 3). The oxygen of the meropenem C6 hydroxyethyl group is positioned to hydrogen bond with the side chain amide NH_2_ of Asn302 (∼3.3 Å) in an analogous manner to the C7 amide oxygen of ceftazidime and cefiderocol (∼2.8 Å), though necessarily meropenem lacks the rest of the C7 interactions made by the cephalosporins, including that of the sidechain amide NH with the alcohol of Thr438. The pyrrolidine linked isopropyl-carboxamide of meropenem is not observed to interact *via* polar interactions with the active site, again contrasting with the cephalosporins, where each structural element, with the exception of the eliminated C3 group, is observed to form interactions in the active site. The potency of PBP3 inhibition by meropenem compared to the more widely interacting cephalosporins is therefore notable.

## Conclusions

Treatment of *P. aeruginosa* infections is a major challenge in control of Gram-negative bacteria, in part due to resistance and in part due to the lack of new and potent antipseudomonal drugs.^22^ Cefiderocol, which was developed as a consequence of long-term research by Shionogi, is a pioneering advance in treatment of pseudomonal infections.^23^ Screening of cefiderocol *versus P. aeruginosa* clinical isolates carrying β-lactamase encoding genes reveals it is highly active against most tested SBL bearing strains.^1,3,23,24^ By comparison, the activities of cefepime and ceftazidime against these strains are substantially weaker, and in some cases, activity was only observed on addition of β-lactamase inhibitors.^22,25^ Cefiderocol has also been shown to be active against some, but not all, metallo-beta-lactamase (MBL) harbouring *P. aeruginosa* clinical isolates.^26–28^

The improved antipseudomonal activity of cefiderocol *versus* other cephalosporins, including cefepime, in MIC testing is proposed to be due to both the ability of cefiderocol to exploit entry through the iron/siderophore transport system, *via* its C3 linked chlorocatechol moiety, and, possibly, because the different C7 side chain increases stability to β-lactamases, especially SBLs.^29^ Interestingly, our pIC_50_ and *k*_inact_/*K*_I_ measurements and meropenem competition experiments with isolated PBP3, imply cefepime (and meropenem) are better PBP3 inhibitors than cefiderocol and ceftazidime, the latter two of which have an identical C7 side chain. Factors other than uptake and potency of PBP3 inhibition are involved in determining overall efficacy (including inhibition of other PBPs) and there is a possibility that the rank order of potency may change in the presence of the natural PBP3 substrates. Nevertheless, the results presented here imply that there is scope for improving the intrinsic antibacterial activity of cefiderocol whilst maintaining its iron/siderophore transport system exploiting ability.

The structural information provided here for the product of the reaction of cefiderocol with *P. aeruginosa* PBP3 will inform on the development of improved antipseudomonal β-lactams. One avenue will be to improve the PBP3 inhibition activity of cefiderocol to match, at least, that observed for cefepime and meropenem. The limited SAR presented here suggests that this should be possible *via* optimisation of the C7 side chain for PBP3 binding, though, this needs to be done in a manner that does not facilitate β-lactamase mediated resistance.

Given the apparently lower number of interactions that meropenem makes with PBP3 compared to the cephalosporins, in particular with respect to the cephalosporin C7 side chain compared to the carbapenem C6 side chain, it is notable that meropenem inhibits PBP3 at least as well as the cephalosporins. Note, using a Bocillin-FL assay for PBP3 under different conditions, Shapiro *et al.* observed more inhibition with ceftazidime than meropenem, though both were potent.^20^ The additional functional groups present in the cephalosporins limit β-lactamase susceptibility and promote binding to the target PBPs. Nonetheless, given that all clinically used carbapenems contain the same C6 (*S*)-configured hydroxyethyl side chain, there would seem to be scope both for optimisation of the existing types of cephalosporin C7 side chains, as well as exploration of different side chains, as is occurring at the analogous position in diazabicyclooctane (DBO) type serine β-lactamase inhibitors and antibacterials.^30–34^ It is also notable that naturally occurring carbapenems have a different group at their C6 position.^35^

Mutations involving iron transport proteins and their regulation which demonstrate resistance to cefiderocol are emerging,^36^ there is thus considerable interest in investigating mutations which confer resistance to cefiderocol specifically those linked to its siderophore group, including to investigate how the nature of the siderophore affects resistance.^24,37–40^ A recent study has shown that cefiderocol resistance in *P. aeruginosa* can be mediated by modifications in genes associated with uptake and efflux, rather than modification of the antibiotic target, PBP3.^41^ Both our MS and crystallographic studies reveal loss of the C3′ groups from the three investigated cephalosporins on reaction with PBP3 (as observed previously for ceftazidime).^14^ Although we cannot be certain of the initial binding modes of the cephalosporins, further SAR at the C3 linked group to optimise PBP3 inhibition may be productive. In future studies, it will be important to consider research that led to the successful development of cefiderocol, for example, the presence of a quaternary amine may be important for efficient uptake into the target bacterium.^42–44^ The catechol group itself must also be carefully selected to avoid toxicity and maintain stability.^45,46^ Given that the siderophore group of cefiderocol is lost on reaction with PBP3, it is also of interest to explore whether this affects resistance, including by using analogues of cefiderocol where such loss cannot occur. Although as yet they have not been developed for clinical use, valuable insight may also come from the development of catechol functionalised monobactams, which likely do not undergo fragmentation on reaction with PBPs.^47–49^

## Materials and methods

### Protein overproduction and purification for pIC_50_ measurements

A gene encoding *ftsI* (PBP3, residues 50–579) from *P. aeruginosa* PAO1 with an N-terminal His_6_-tag was inserted into a pET-28a(+) vector (using NcoI to HindIII sites) (obtained from GenScript). BL21(DE3) cells (New England Biolabs) were transformed with the plasmid *via* heat shock (42 °C, 45 seconds), 2 YT media (200 μL) added and the mixture grown (1 hour, 37 °C), then plated onto 2 YT agar (50 μg mL^−1^ kanamycin) and incubated overnight (37 °C). A single colony was selected and grown overnight in 2 YT medium (100 mL, 37 °C, 200 rpm, 50 μg mL^−1^ kanamycin). Terrific broth (TB) autoinduction medium (Formedium, 6 L, 50 μg mL^−1^ kanamycin) was inoculated with an overnight culture (6 mL per 1 L of terrific broth) and incubated overnight (24 hours, 25 °C, 180 rpm). Cells were harvested *via* centrifugation (8000 rpm, 10 minutes), then stored at – 80 °C.

Cells were thawed and resuspended in buffer (25 mM Tris, 400 mM NaCl, 10 mM Imidazole, 10% glycerol, pH 8.0, 0.1% CHAPS, 100 μg DNase, 1 cOmplete mini EDTA-free protease inhibitor tablet, Roche). Cells were lysed *via* cell disruption (25 kPsi, Constant Systems Continuous Flow Cell Disruptor). The cell debris was pelleted *via* centrifugation (22 000 rpm, 30 minutes, 4 °C). The supernatant was filtered (0.45 μM) and loaded onto a Ni-NTA column (5 mL, GE Healthcare, flow rate 2.5 mL min^−1^). The column was washed with resuspension buffer (15 CV); the protein was eluted with a stepwise gradient of imidazole; 50 mM (5 CV), 100 mM (3 CV), 200 mM (3 CV), 300 mM (3 CV), 500 mM (3 CV). Gel electrophoresis (180 V, 45 min) was carried out using a 4–12% NuPAGE gel (Invitrogen, ESI Fig. S9†) to identify fractions containing sufficiently purified PBP3. Such fractions were pooled, concentrated, and buffer exchanged using a PD-10 column into 25 mM Tris, 400 mM NaCl, 10%_v/v_ glycerol, pH 8.0.

### Recombinant PBP3 production and purification for crystallography

For crystallographic studies, DNA encoding PBP3 (residues 53–562) was cloned into a pRSETa vector (Invitrogen) modified to include a N-terminally His_6_-tagged lipoyl domain from the *Bacillus stearothermophilus* dihydrolipoamide acetyltransferase domain, with a C-terminal TEV protease cleavage site (Invitrogen). The cloning procedure resulted in incorporation of four additional residues at the C-terminus of PBP3, which serendipitously resulted in improved diffraction compared to our original construct which matched that from the literature (ESI Fig. S3 and S4†).^14^ In some structures the additional residues appear to form an intramolecular contact between asymmetric units within the unit cell, possibly reflecting the improved diffraction of the truncated construct compared to the full length construct lacking the serendipity tag (ESI, Fig. S5†).

The construct used for crystallography was expressed in *E. coli* C41(DE3) cells. Once transformed with the pRSETa vector, these were grown in 2-YT media at 37 °C and, once the OD_600_ exceeded 0.8, protein production was induced *via* the addition of isopropyl β-d-1-thiogalactopyranoside (IPTG) to a final concentration of 1 mM. Following overnight incubation (18 °C), cells were harvested *via* centrifugation and resuspended in buffer A (20 mM Tris, pH 8.0, 300 mM NaCl, 20 mM imidazole, 5 mM 2-mercaptoethanol). Cells were lysed *via* sonication. Cell debris was subsequently removed *via* centrifugation (20 000 rpm, 40 min, 4 °C). The protein was then purified *via* Ni-affinity chromatography using a gradient profile. The elution buffer (Buffer B) contained 20 mM Tris, pH 8.0, 300 mM NaCl, 500 mM imidazole and 5 mM 2-mercaptoethanol. Fractions containing PBP3 were pooled and digested overnight with a His-tagged TEV protease at a final concentration of 0.1 mg mL^−1^. The protein was then dialysed overnight (4 °C) into 20 mM Tris, pH 8.0, 300 mM NaCl. The digested PBP3 was reapplied to the Ni-affinity column, which had been pre-equilibrated with buffer A. The fractions containing highly purified PBP3 were collected, whilst undigested PBP3, His-tagged lipoyl domain and TEV protease were retained on the column. The protein was concentrated to 1.8 mg mL^−1^ and dialysed into 10 mM Tris, pH 8.0, 100 mM NaCl for crystallography. PBP3 was crystallised at 4 mg mL^−1^ by sitting-drop vapour diffusion. Co-crystallisation was achieved by incubation of PBP3 with inhibitor (0.5 mM) for ten minutes prior to setup of the crystal trays. Several conditions yielded crystals with SG1 D2, JCSG A8, PACT B12 and PACT H8 conditions being used to obtain datasets (ESI Table S1†).

X-ray data were collected at beamline I03 at Diamond Light Source (Harwell, UK). Diffraction data were indexed and integrated using DIALS.^50,51^ Crystals were in the *P*2_1_2_1_2_1_ or *P*2_1_ space groups, with one (*P*2_1_2_1_2_1_) or two (*P*2_1_) molecules per asymmetric unit. Each structure was solved *via* molecular replacement using Phaser.^52^ An AlphaFold2 model of *P. aeruginosa* PBP3 was used as the starting template.^53^ Multiple rounds of refinement were undertaken using PHENIX with manual model building undertaken using COOT.^54,55^ This process was repeated until *R*_free_ and *R*_work_ converged. The statistics for the final, refined structures are provided (ESI Table S1†).

### S2d pIC_50_ measurements

A modified procedure based on that of López-Pérez *et al.* was used.^17^ For measurement of pIC_50_ values using the S2d probe,^56^*P. aeruginosa* PBP3 (300 nM) in S2d assay buffer (25 mM HEPES, 100 mM NaCl, pH 7.4) was incubated with cefiderocol, cefepime, or ceftazidime (100 μM to 5 nM, ten concentrations total, 3-fold dilution series) for 10 minutes at 30 °C, 300 rpm. A positive control without inhibitor and a negative control lacking both enzyme and inhibitor were prepared. In both cases the inhibitor/enzyme was replaced with buffer. Following incubation of the inhibitor with PBP3, S2d probe (1.5 mM) and monobromobimane (mBBr, 0.5 mM) were dispensed to initiate reaction. The increase in fluorescence intensity of the mBBr (*λ*_Ex_/*λ*_Em_ = 394/490 nm) was monitored using a BMG CLARIOstar Plus Microplate Reader using black non-binding 384-well microplates (Greiner). The initial reaction rate was calculated for each compound concentration by fitting to a linear regression and these were used to calculate relative pIC_50_ values using the log([inhibitor]) against normalised response fit using GraphPad Prism version 10.2 (ESI Fig. S6†).

### Bocillin-FL pIC_50_ measurements

For measurement of pIC_50_ values using Bocillin-FL a modified procedure based on that of López-Pérez *et al.* was used.^17,18^*P. aeruginosa* PBP3 (311 nM) in Bocillin-FL assay buffer (50 mM potassium phosphate, pH 7.4) was incubated with cefiderocol, cefepime, or ceftazidime (100 μM to 5 nM; ten concentrations with 3-fold dilutions) for 10 min (30 °C, 300 rpm). A positive control without inhibitor and a negative control lacking both enzyme and inhibitor were prepared. In both cases the inhibitor/enzyme solution was replaced with an equal volume of buffer. Following incubation of the inhibitor with PBP3, Bocillin-FL (30 nM) was dispensed to initiate the reaction. Following a further incubation (10 min, 30 °C, 300 rpm) the fluorescence polarisation was measured using a BMG PHERAstar Microplate Reader (*λ*_Ex_/*λ*_Em_ = 480/520 nm) using black 384-well microplates (Greiner). The percentage inhibition of Bocillin-FL binding was calculated for each compound concentration by normalising with control wells. These measurements were used to calculate relative pIC_50_ values using the log([inhibitor]) against normalised response, fit, using GraphPad Prism version 10.2 (ESI Fig. S7†).

### Bocillin-FL *k*_inact_/*K*_i_ measurements

For measurement of *k*_inact_/*K*_i_ values using Bocillin-FL a modified procedure based on that of Shapiro *et al.* was used.^20^*P. aeruginosa* PBP3 (60 nM) in Bocillin-FL assay buffer (50 mM potassium phosphate, pH 7.4) was added to a mixture of cefiderocol, cefepime or ceftazidime (20 μM to 39 nM, ten concentrations total with 3-fold dilutions) with Bocillin-FL (30 nM). A positive control without inhibitor and a negative control lacking both enzyme and inhibitor were prepared. In both cases the inhibitor/enzyme solution was replaced with an equal volume of buffer. Immediately following mixing, the change in fluorescence polarisation was measured using a BMG PHERAstar Microplate Reader (*λ*_Ex_/*λ*_Em_ = 480/520 nm) using black 384-well microplates (Greiner). Progress curves for all compound concentrations were analysed and fit using KinTek Global Kinetic Explorer Version v11.0.1 (KinTek, Austin, TX, USA, ESI Fig. S8†) according to the procedure detailed by Shapiro *et al*.^20^ in brief, the simplified model used to fit the data was:
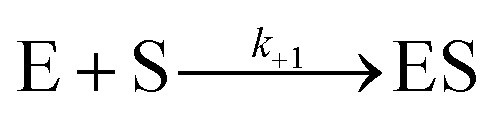

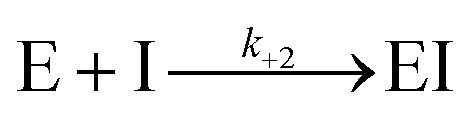
where E represents PBP3, S represents Bocillin-FL and I the inhibitor tested. The observable was defined as:Offset + scale × [ES]

The offset was defined as the polarisation value of the negative control. [ES] is the concentration of the PBP-Bocillin-FL complex at each time point and the scale represents the scaling factor which relates the increase in fluorescence polarisation to the concentration of the PBP-Bocillin-FL complex formed.^20^ The reverse reactions were negligible in both cases and inclusion of a non-zero value for *k*_−1_ or *k*_−2_ did not improve the fit of the data, consequently these parameters were omitted. *k*_+2_ is equivalent to *k*_inact_/*K*_i_.

### Meropenem competition binding experiments

Protein samples (100 μM) were preincubated with a 1 : 1 mixture of a cephalosporin and meropenem (1 mM of each) for ten minutes before quenching *via* addition of formic acid (1%_v/v_). Samples were then diluted in LC-MS-grade water (15 μM final concentration) and analysed using a Waters XeVo G2-S mass spectrometer coupled to a Waters Acquity-UPLC. 5 μL samples were injected onto a ThermoFisher Scientific ProSwift RP-4H (1 mm × 50 mm) column pre-equilibrated in 95%_v/v_ water, 5%_v/v_ MeCN. The column was eluted with a linear gradient from 5 to 95_v/v_% MeCN in water over ten minutes. All solvents contained 0.1%_v/v_ formic acid. Data were analysed using MassLynx Version 4.1. The mass spectra obtained were deconvoluted using the MaxEnt1 algorithm.

### Protein observed ESI-Q-TOF mass spectrometry

Protein samples (100 μM) for MS analyses were preincubated with inhibitors (2 mM) for 10 minutes before dilution in LC-MS-grade water to a final concentration of 10 μM. Samples were analysed using a Waters XeVo G2-S mass spectrometer coupled to a Waters Acquity-UPLC. Samples (5 μL) were injected onto a ThermoFisher Scientific ProSwift RP-4H (1 mm × 50 mm) column pre-equilibrated in 95%_v/v_ water, 5%_v/v_ MeCN. The column was eluted with a linear gradient from 5 to 95%_v/v_ ACN in water over ten minutes. All solvents contained 0.1%_v/v_ formic acid. Data were analysed using MassLynx Version 4.1. The mass spectra obtained were deconvoluted using the MaxEnt1 algorithm.

## Data availability

Crystallographic data has been deposited at the PDB under accession codes 9FZ7, 9FZ8, 9FZO, 9FZP and 9FZE. Additional figures and tables can be found in the ESI.† Requests for data should be made to Christopher J. Schofield (christopher.schofield@chem.ox.ac.uk).

## Author contributions

H. G. S. conceptualization, methodology, analysis, investigation, writing. S. B. and V. A. production of S2d probe. M. J. B. methodology, analysis. F. M. A. investigation. M. D. A. methodology, investigation, analysis. A. J. M. F. conceptualization, supervision. C. J. S. conceptualization, methodology, supervision, writing.

## Conflicts of interest

The authors have no conflicts to declare.

## Supplementary Material

SC-OLF-D4SC04937C-s001
