# Supplementary material for: Structural basis of Pseudomonas aeruginosa penicillin binding protein 3 inhibition by the siderophore-antibiotic cefiderocol
Source: Chem Sci. 2024 Sep 18;15(41):16928–37. doi: 10.1039/d4sc04937c (PMC11423509; doi:10.1039/d4sc04937c)
Supplement: SC-015-D4SC04937C-s001 [file SC-015-D4SC04937C-s001.pdf]

Supplementary Information (SI) for Chemical Science.
This journal is © The Royal Society of Chemistry 2024

Structural basis of *Pseudomonas aeruginosa* penicillin binding protein 3 inhibition by the siderophore antibiotic cefiderocol

Helen G. Smith,[a,b] Shyam Basak,[a,b] Victor Aniebok,[a,b] Matthew J. Beech,[a,b] Faisal M. Alshref,[a,c] Mark D. Allen,[a,b] Alistair J.M. Farley,[a,b] and Christopher J. Schofield[a,b]

[a] Department of Chemistry, University of Oxford, 12 Mansfield Road, Oxford, OX1 3TA, United Kingdom.

[b] Ineos Oxford Institute for Antimicrobial Research, University of Oxford, 12 Mansfield Road, Oxford, OX1 3TA, United Kingdom.

[c] Department of Biochemistry, Faculty of Science, King AbdulAziz University, Jeddah, Saudi Arabia.

Supplementary Information

1. **Figure S1:** Ligplot+ schematics displaying contacts formed between: (**A**) cefiderocol (PDB: **9FZ7**), (**B**) ceftazidime (PDB: **9FZO**), (**C**) cefepime (PDB: **9FZP**) and (**D**) meropenem (PDB: **9FZE**) reaction products and residues within the *P. aeruginosa* PBP3 active site.
2. **Figure S2:** Interaction map displaying polar interactions formed between (**A**) cefiderocol (PDB: **9FZ7**), (**B**) ceftazidime (PDB: **9FZO**), (**C**) cefepime (PDB: **9FZP**) and (**D**) meropenem (PDB: **9FZE**) reaction products and residues within the *P. aeruginosa* PBP3 active site.
3. **Figure S3:** Superimposition of structural views of apo *P. aeruginosa* PBP3 (orange, PDB: **9FZ8**) and PBP3 following reaction with: ceftazidime (blue, PDB: **9FZO**), cefepime (green, PDB: **9FZP**), cefiderocol (grey, PDB: **9FZ7**), and meropenem (pink, PDB: **9FZE**).
4. **Figure S4:** View from a crystal structure of *P. aeruginosa* PBP3 (9FZ8.pdb) coloured grey and tan) showing the interface and contacts formed by the C-terminal 'serendipity'-tag (coloured green; residues: EFEA) between each protein molecule within adjacent unit cells.
5. **Figure S5:** Evidence reaction of *P. aeruginosa* PBP3 with pyrrolidinium cephalosporins occurs with loss of the C3 side chain.
6. **Scheme S1:** The reaction of the S2d substrate analogue with PBP3.
7. **Scheme S2:** The reaction of Bocillin-FL, a BODIPY-labelled penicillin derivative, with PBP3.
8. **Figure S6:** Dose response curves for (**A**) cefiderocol, (**B**) ceftazidime, (**C**) cefepime and (**D**) meropenem with *P. aeruginosa* PBP3 measured using S2d assays.
9. **Figure S7:** Dose response curves for (**A**) cefiderocol, (**B**) ceftazidime, (**C**) cefepime and (**D**) meropenem with *P. aeruginosa* PBP3 measured using Bocillin-FL assays.
10. **Figure S8:** Representative progress curves for (**A**) cefiderocol, (**B**) ceftazidime, (**C**) cefepime and (**D**) meropenem with *P. aeruginosa* PBP3 measured using Bocillin-FL assays.
11. **Figure S9:** SDS-PAGE gel of purified *P. aeruginosa* PBP3 and deconvoluted mass spectrum of the purified protein.
12. **Table S1:** Crystallography data collection and refinement statistics for apo *P. aeruginosa* PBP3 and following reaction with beta-lactams.
13. **Table S2:** RMSD C-α values calculated for *P. aeruginosa* PBP3 crystal structures.

14. **References**.

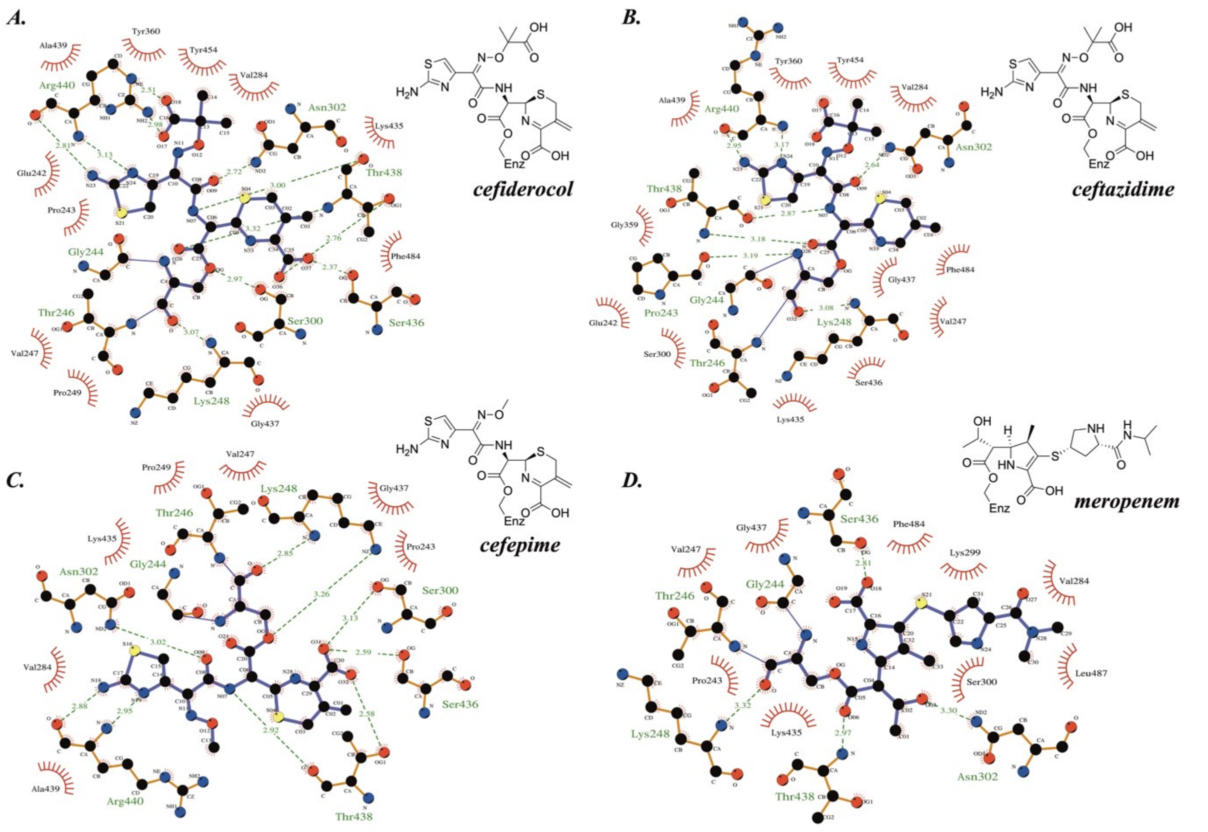

Figure S1: Ligplot+ schematics displaying contacts formed between: (A) cefiderocol (PDB: 9FZ7), (B) ceftazidime (PDB: 9FZO), (C) cefepime (PDB: 9FZP) and (D) meropenem (PDB: 9FZE) reaction products and residues within the *P. aeruginosa* PBP3 active site. The figure was generated using LigPlot+ version 2.2.

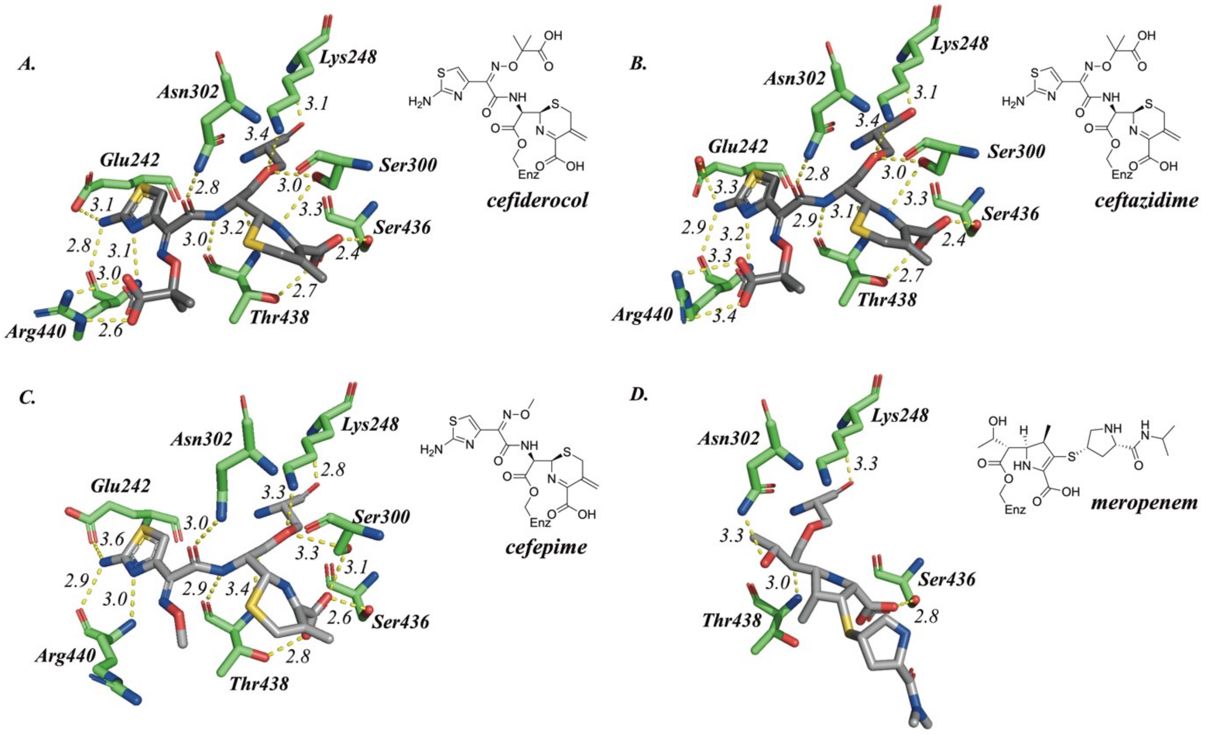

Figure S2: Interaction map displaying polar interactions formed between (A) cefiderocol (PDB: 9FZ7), (B) ceftazidime (PDB: 9FZO), (C) cefepime (PDB: 9FZP) and (D) meropenem (PDB: 9FZE) reaction products and residues within the *P. aeruginosa* PBP3 active site. Cephalosporin derived atoms are coloured grey; interacting residues are green. Interaction distances are in Å. The figure was generated using Pymol Version 2.5.4 (Schrödinger LLC).[1] Note the acyl-enzyme complex derived from meropenem was refined as the enamine tautomer, which is the major apparent species, though the presence of low levels of imine tautomers cannot be ruled out (based on work with serine β-lactamases).[2] A structure of *P. aeruginosa* PBP3 with meropenem bound has been previously reported.[3]

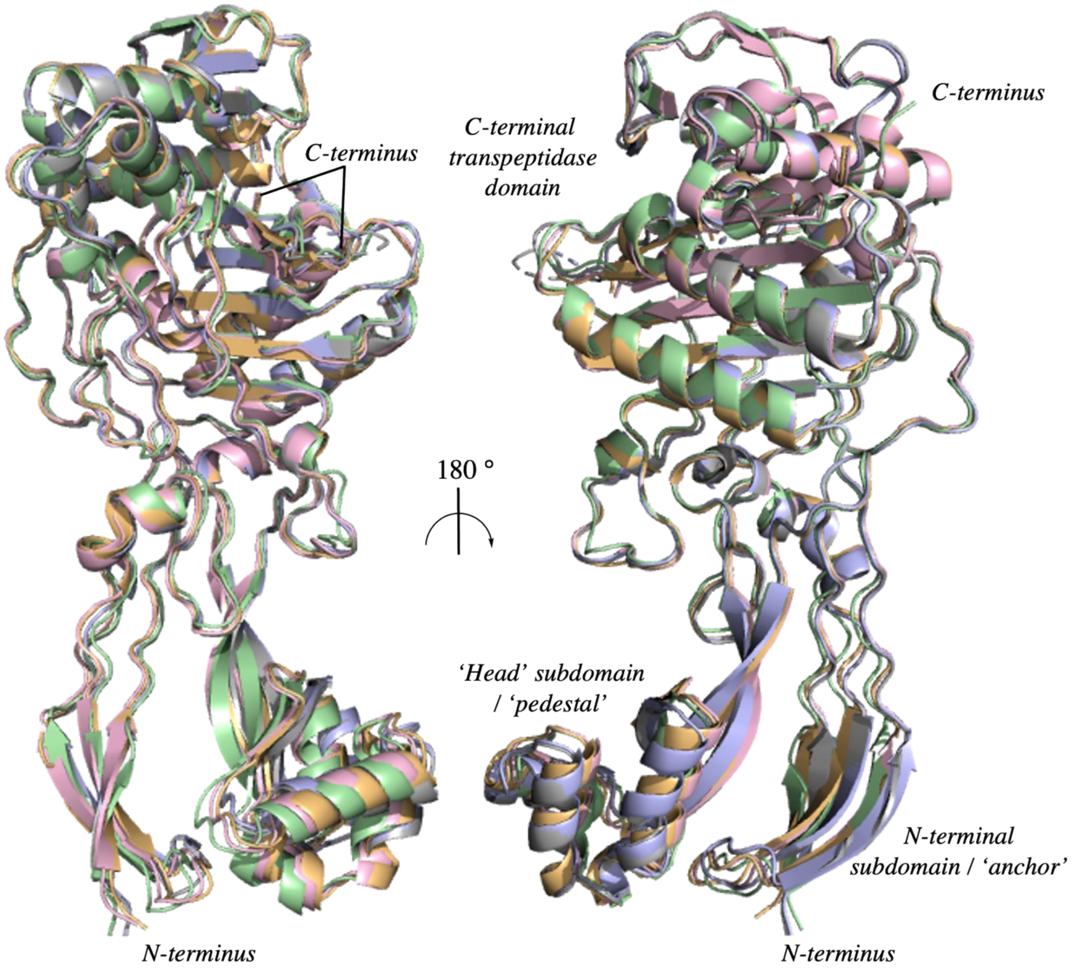

Figure S3: Superimposition of structural views of apo *P. aeruginosa* PBP3 (orange, PDB: 9FZ8) and PBP3 following reaction with: ceftazidime (blue, PDB: 9FZO), cefepime (green, PDB: 9FZP), cefiderocol (grey, PDB: 9FZ7), and meropenem (pink, PDB: 9FZE). The figure was generated using Pymol Version 2.5.4.

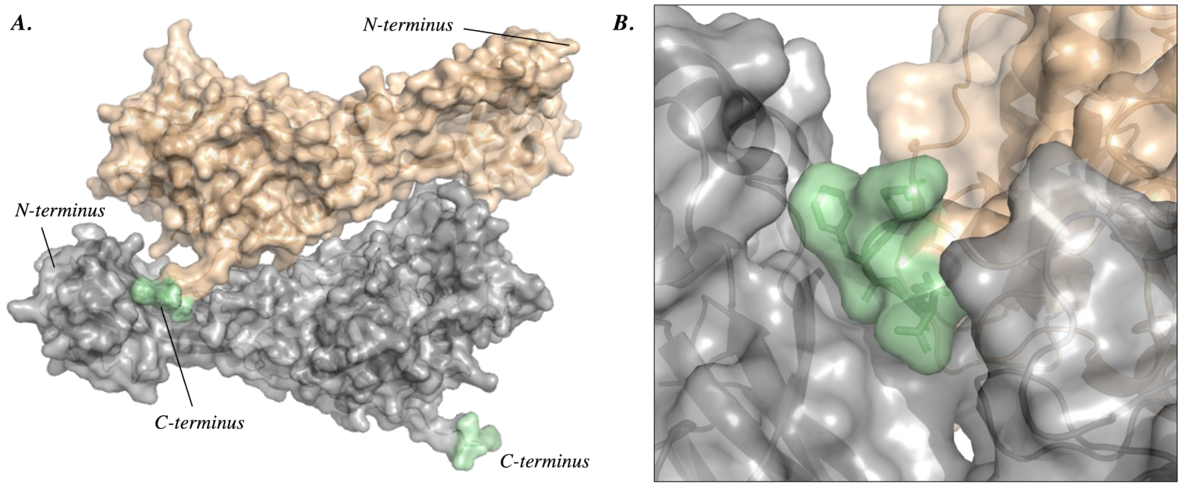

Figure S4: Figure S4: View from a crystal structure of *P. aeruginosa* PBP3 (9FZ8.pdb) coloured grey and tan) showing the interface and contacts formed by the C-terminal 'serendipity'-tag (coloured green; residues: EFEA) between each protein molecule within adjacent unit cells. It is proposed that the contacts that this tag forms act as a stabilising interaction across the crystal lattice possibly relating to the improvement in the diffraction observed relative to the construct without the tag (from 2.5 – 3.5 Å to < 2.5 Å in most cases).

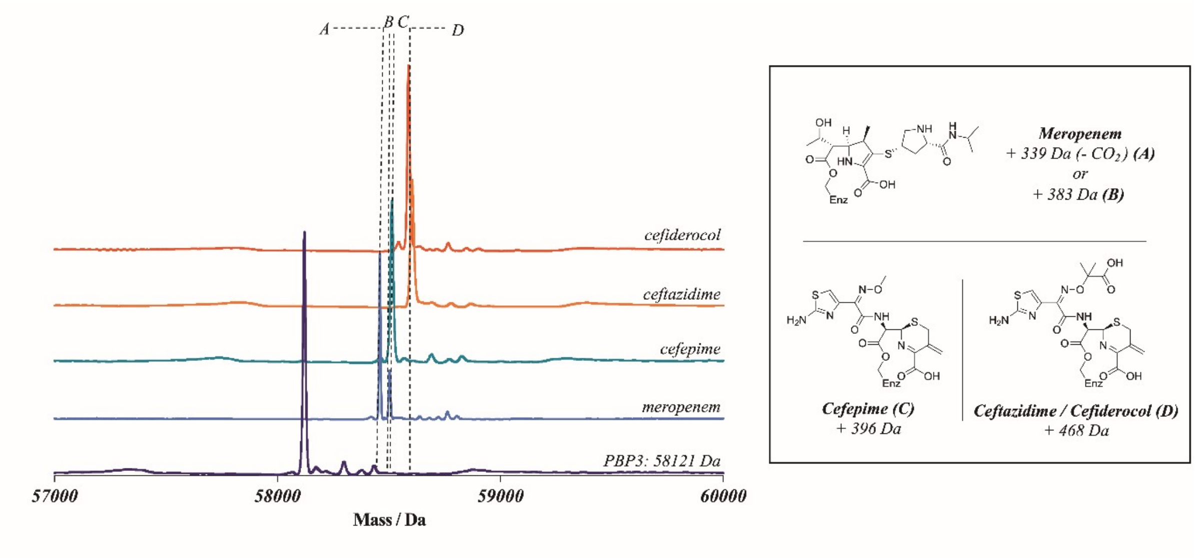

Figure S5: **Evidence reaction of *P. aeruginosa* PBP3 with pyrrolidinium cephalosporins occurs with loss of the C3 side chain.** Deconvoluted electrospray-ionisation Quadrupole Time-of-Flight (ESI-Q-TOF) mass spectra following reaction of *P. aeruginosa* PBP3 with β-lactams gives rise to new peaks with meropenem (**A** & **B**), cefepime (**C**) and ceftazidime / cefiderocol (**D**). See Methods Section for experimental details. The spectra shown are representative of two technical replicates. Observed mass of unmodified PBP3 (- N-terminal Met): 58,121 Da (calculated 58,121 Da). Note decarboxylation of meropenem during electrospray ionisation has been previously reported.[4]

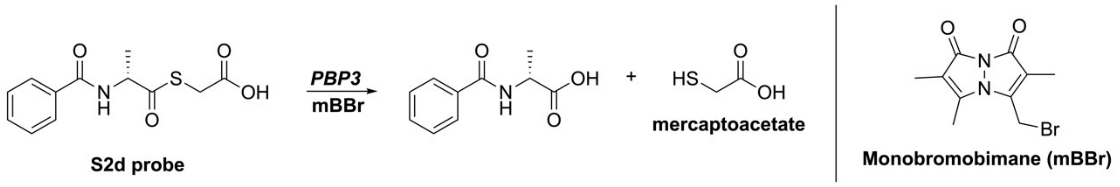

Scheme S1: The reaction of the S2d substrate analogue with PBP3. The reaction product mercaptoacetate contains a free thiol, which is reacted with monobromobimane (mBBr) producing a fluorescent response, allowing reaction progress to be monitored.[5]

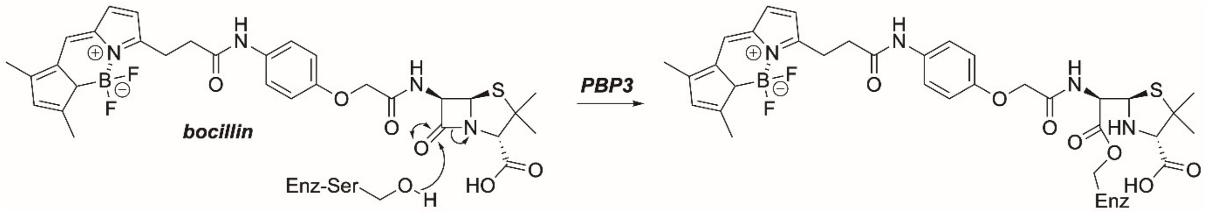

Scheme S2: The reaction of Bocillin-FL, a BODIPY-labelled penicillin derivative, with PBP3. Formation of the covalent acyl-enzyme complex is monitored by measuring change in fluorescence polarisation which manifests on β-lactam reaction.[5,6]

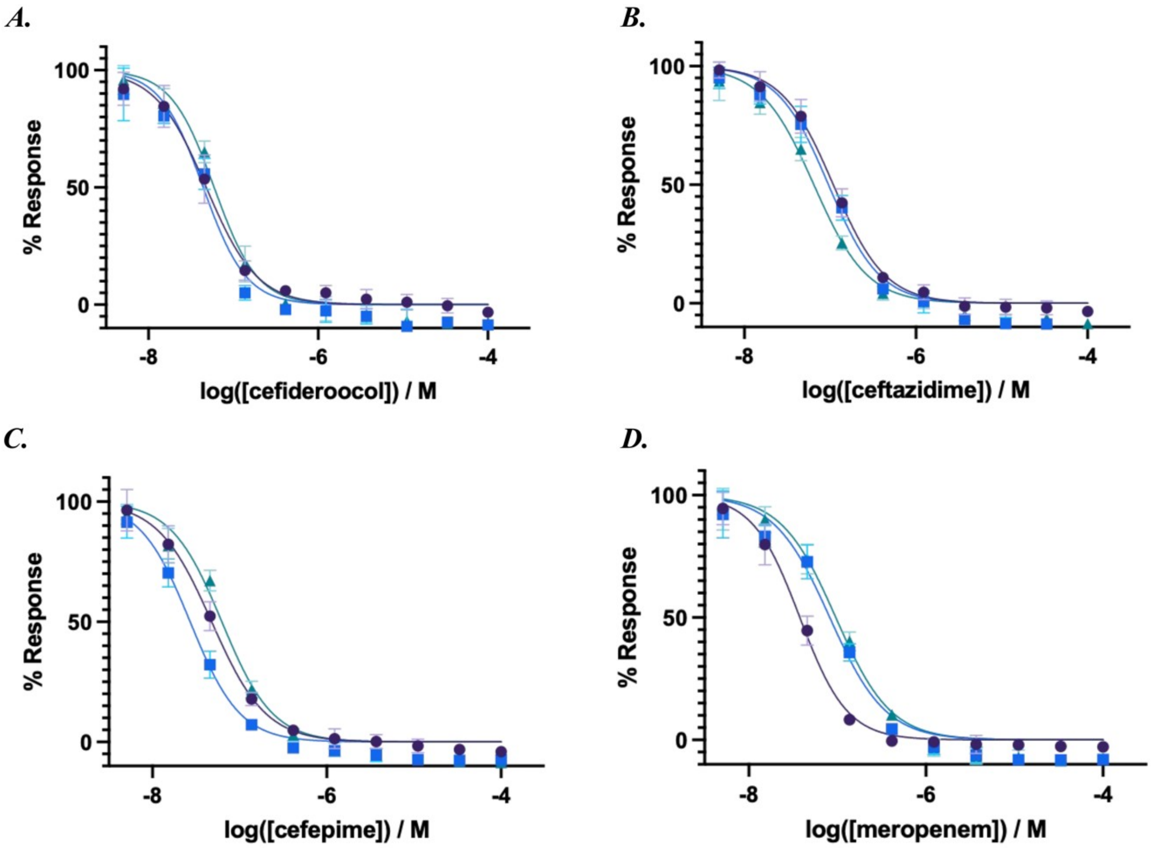

Figure S6: Dose response curves for (A) cefiderocol, (B) ceftazidime, (C) cefepime and (D) meropenem with *P. aeruginosa* PBP3 measured using S2d assays. Inhibition assays were carried out with 300 nM PBP3, 1.5 mM S2d and 0.5 mM mBBr, with 10 minutes pre-incubation with inhibitor compounds in 25 mM HEPES, 100 mM NaCl, pH 7.4. Three independent repeats are shown. Error bars represent standard deviations from three technical replicates measured simultaneously. Average pIC_{50} values and inhibitor structures are provided in **Table 1**. The S2d probe was synthesised according to a literature procedure.[7]

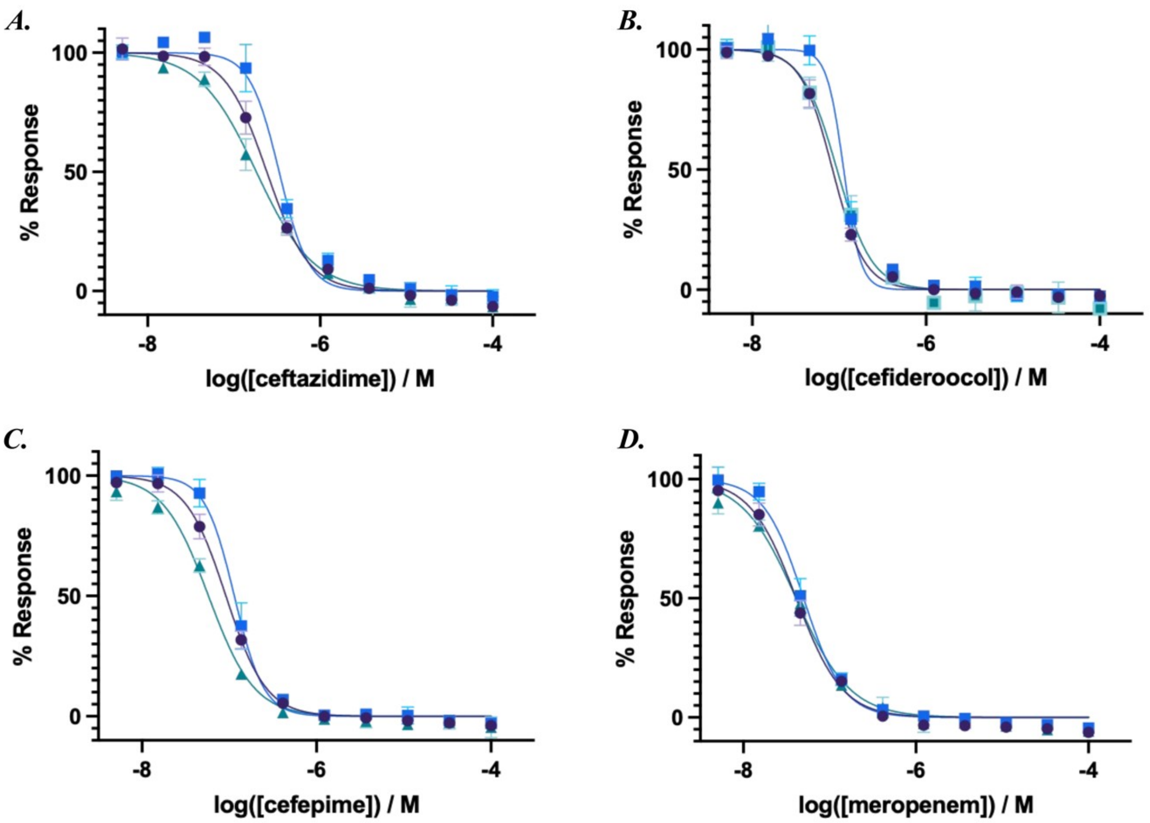

Figure S7: Dose response curves for (A) cefiderocol, (B) ceftazidime, (C) cefepime and (D) meropenem with *P. aeruginosa* PBP3 measured using Bocillin-FL assays. Inhibition assays were carried out with 311 nM PBP3 and 30 nM Bocillin-FL, with 10 minute pre-incubation with inhibitor compounds in 50 mM potassium phosphate, pH 7.4. Three independent repeats are shown. Error bars represent standard deviations from three technical replicates measured simultaneously. Average pIC_{50} values and inhibitor structures are provided in **Table 1**.

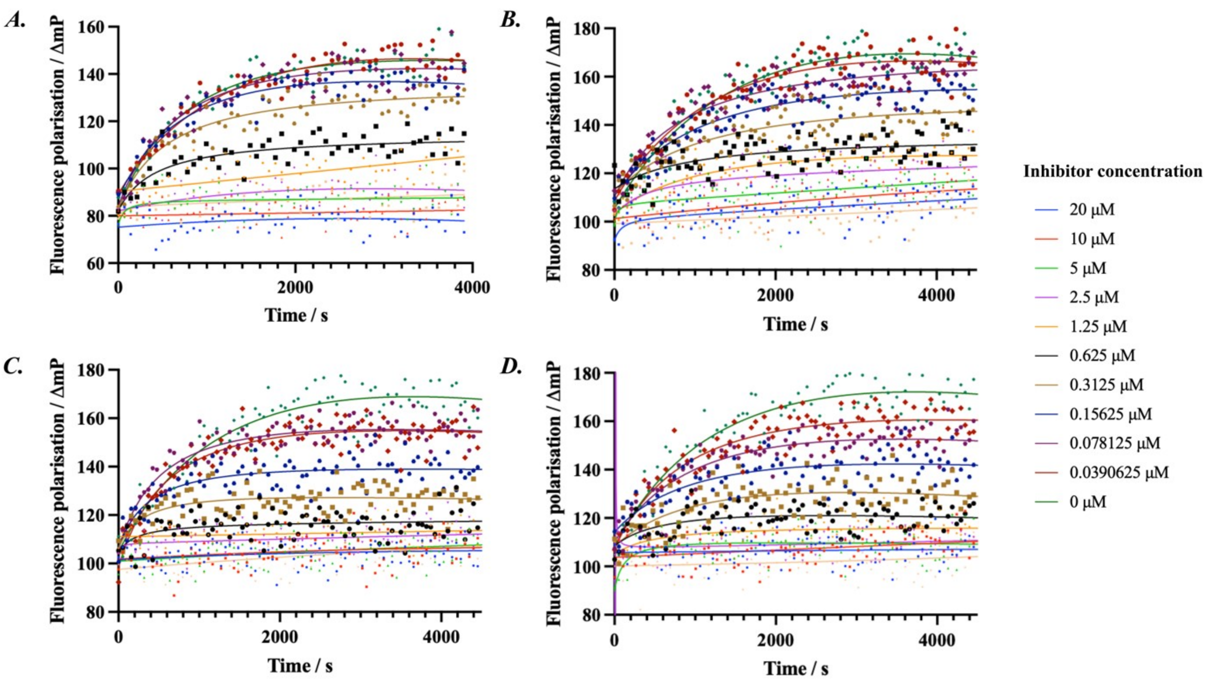

Figure S8: Representative progress curves for (A) cefiderocol, (B) ceftazidime, (C) cefepime and (D) meropenem with *P. aeruginosa* PBP3 measured using Bocillin-FL assays. Inhibition assays were carried out with 60 nM PBP3 and 30 nM Bocillin-FL in 50 mM potassium phosphate, pH 7.4, buffer. Three independent repeats were undertaken. Fitting was carried out using KinTek Global Kinetic Explorer Version v11.0.1 (KinTek, Austin, TX, USA) according to the procedure detailed by Shapiro *et. al*.[8,9] Average k_{inact}/K_i values and inhibitor structures are provided in **Table 1**.

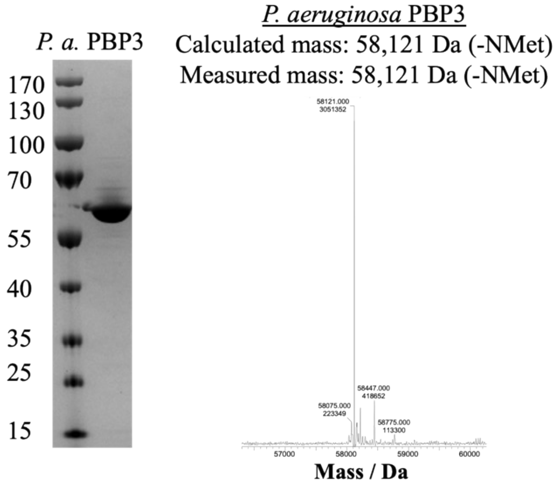

Figure S9: SDS-PAGE gel of purified *P. aeruginosa* PBP3 and deconvoluted mass spectrum of the purified protein.

Table S1: Crystallography data collection and refinement statistics for apo *P. aeruginosa* PBP3 and following reaction with beta-lactams.[10–14]

Data set	apo-PBP3	Cefiderocol	Ceftazidime	Cefepime	Meropenem
Wavelength (Å)	0.97626	0.97626	0.97626	0.97626	0.97626
Resolution range (Å)	54.14 – 2.11	61.07 – 1.80	54.39 – 1.80	53.59 – 2.70	60.89 – 2.10
Unique reflections[a]	30196 (2387)	48820 (2358)	47543 (2299)	12157 (1620)	30572 (2486)
Completeness (%)	99.8 (98.1)	99.9 (98.0)	98.7 (98.0)	87.8 (89.3)	100 (100)
*R*merge[b]	0.146 (1.331)	0.093 (1.068)	0.105 (1.000)	0.299 (1.529)	0.170 (1.246)
*R*pim[a]	0.046 (0.416)	0.026 (0.306)	0.038 (0.393)	0.108 (0.558)	0.055 (0.398)
CC(1/2)[c]	0.998 (0.815)	0.998 (0.812)	0.997 (0.612)	0.987 (0.546)	0.998 (0.782)
Multiplicity[d]	10.8 (11.1)	13.6 (12.9)	8.6 (7.4)	7.8 (8.0)	10.5 (10.7)
I/σI[1]	6.9 (1.8)	15.9 (1.1)	12.4 (1.9)	4.2 (1.1)	9.0 (1.9)
Space group	$P2_12_12_1$	$P2_12_12_1$	$P2_12_12_1$	$P2_12_12_1$	$P2_12_12_1$
Unit cell parameters	a = 67.92 Å, b = 84.01 Å, c = 89.54 Å	a = 69.05 Å, b = 82.96 Å, c = 90.22 Å	a = 68.33 Å, b = 83.18 Å, c = 89.89 Å	a = 67.30 Å, b = 88.47 Å, c = 82.01 Å	a = 68.70 Å, b = 89.08 Å, c = 83.45 Å
**Model refinement**					
Resolution range (Å)	45.49 – 2.11	45.11 – 1.80	45.53 – 1.80	53.56 – 2.70	44.54 – 2.10
No. of reflections (working/free)	30063 (2793)	48724 (1986)	47304 (2374)	12048 (690)	29783 (2849)
No. of residues	A, 1 - 512	A, 1 - 517	A, 1 - 517	A, 3 - 513	A, 1 - 512
No. of water, ligand molecules	215	503	630	33	344
*R*work/*R*free,[e] %	0.226 / 0.263	0.189 / 0.218	0.187 / 0.222	0.250 / 0.296	0.213 / 0.254
B average[f]	34.81 Å	33.98 Å	26.10 Å	51.33 Å	29.28 Å
Geometry bonds / angles[g]	0.002 Å, 0.49 °	0.004 Å, 0.66 °	0.004 Å, 0.68 °	0.006 Å, 1.20 °	0.002 Å, 0.49 °
Ramachandran[h]	96.4 % / 0.0 %	98.4 % / 0.0 %	98.2 % / 0.0 %	97.0 % / 0.0 %	96.2 % / 0.4 %
PDB ID[i]	**9FZ8**.pdb	**9FZ7**.pdb	**9FZO**.pdb	**9FZP**.pdb	**9FZE**.pdb

[a]Statistics of highest resolution bin in brackets. [b]R_m: $\Sigma_h\Sigma_i|I(h,i)-I(h)| / \Sigma_h\Sigma_i I(h,i)$ where I(h,i) are symmetry related intensities and I(h) is the mean intensity of the reflection with unique index h. [c]$CC_{1/2}$ is the correlation coefficient of the mean intensities between two random half-datasets. [d]Multiplicity for unique reflections. [e]5% of reflections were randomly selected for determination of the free R-factor, prior to any refinement. [f]Temperature factors averaged for

all atoms. [g]RMS deviations from ideal geometry for bond lengths and restraint angles (Engh and Huber). [h]Percentage of residues in the ‘most favoured region’ of the Ramachandran plot and percentage of outliers (PROCHECK). [i]Protein Data Bank identifiers for co-ordinates.

Table S2: RMSD C-α values calculated for *P. aeruginosa* PBP3 crystal structures. Calculated using Pymol Version 2.5.4.

	**Apo-PBP3**	**Cefepime**	**Ceftazidime**	**Cefiderocol**	**Meropenem**
**Apo-PBP3**	-	1.02 Å	1.07 Å	1.04 Å	0.29 Å
**Cefepime**	1.02 Å	-	1.31 Å	1.24 Å	0.96 Å
**Ceftazidime**	1.07 Å	1.31 Å	-	0.23 Å	1.12 Å
**Cefiderocol**	1.04 Å	1.24 Å	0.23 Å	-	1.09 Å
**Meropenem**	0.29 Å	0.96 Å	1.12 Å	1.09 Å	-

References

1 R. A. Laskowski and M. B. Swindells, *J. Chem. Inf. Model*, 2011, **51**, 2778–2786.

2 C. T. Lohans, E. I. Freeman, E. van Groesen, C. L. Tooke, P. Hinchliffe, J. Spencer, J. Brem and C. J. Schofield, *Sci. Rep.*, 2019, **9**, 13608.

3 S. Han, R. P. Zaniewski, E. S. Marr, B. M. Lacey, A. P. Tomaras, A. Evdokimov, J. R. Miller and V. Shanmugasundaram, *Proc. Natl. Acad. Sci. U.S.A.*, 2010, **107**, 22002–22007.

4 T. A. Zandi and C. A. Townsend, *Proc. Natl. Acad. Sci. U.S.A.*, 2021, **118**, e2008610118.

5 A. López-Pérez, S. Freischem, I. Grimm, O. Weiergräber, A. Dingley, M. López-Alberca, H. Waldmann, W. Vollmer, K. Kumar and C. Vuong, *Antibiotics*, 2021, **10**, 529.

6 G. Zhao, T. I. Meier, S. D. Kahl, K. R. Gee and L. C. Blaszczak, *Antimicrob. Agents Chemother.*, 1999, **43**, 1124–1128.

7 J. F. Simon, A. Bouillez, J.M. Frère, A. Luxen and A. Zervosen, *Arkivoc*, 2016, **5**, 22–31.

8 A. B. Shapiro, J. Comita-Prevoir and M. Sylvester, *ACS Infect. Dis.*, 2019, **5**, 863–872.

9 A. B. Shapiro, R. F. Gu, N. Gao, S. Livchak and J. Thresher, *Anal. Biochem.*, 2013, **439**, 37–43.

10 D. Liebschner, P. V. Afonine, M. L. Baker, G. Bunkóczi, V. B. Chen, T. I. Croll, B. Hintze, L. W. Hung, S. Jain, A. J. McCoy, N. W. Moriarty, R. D. Oeffner, B. K. Poon, M. G. Prisant, R. J. Read, J. S. Richardson, D. C. Richardson, M. D. Sammito, O. V. Sobolev, D. H. Stockwell, T. C. Terwilliger, A. G. Urzhumtsev, L. L. Videau, C. J. Williams and P. D. Adams, *Acta Crystallogr. D Struct. Biol.*, 2019, **75**, 861–877.

11 P. Emsley, B. Lohkamp, W. G. Scott and K. Cowtan, *Acta Crystallogr. D Biol. Crystallogr.*, 2010, **66**, 486–501.

12 G. Winter, D. G. Waterman, J. M. Parkhurst, A. S. Brewster, R. J. Gildea, M. Gerstel, L. Fuentes-Montero, M. Vollmar, T. Michels-Clark, I. D. Young, N. K. Sauter and G. Evans, *Acta Crystallogr. D Struct. Biol.*, 2018, **74**, 85–97.

13 A. J. McCoy, R. W. Grosse-Kunstleve, P. D. Adams, M. D. Winn, L. C. Storoni and R. J. Read, *J. Appl. Crystallogr.*, 2007, **40**, 658–674.

14 G. Winter, J. Beilsten-Edmands, N. Devenish, M. Gerstel, R. J. Gildea, D. McDonagh, E. Pascal, D. G. Waterman, B. H. Williams and G. Evans, *Protein Sci.*, 2022, **31**, 232–250.
